# Rheumatoid Arthritis and Heart Failure: A Narrative Review

**DOI:** 10.7759/cureus.74238

**Published:** 2024-11-22

**Authors:** Korimerla Deepika, Sai Lokesh Moraboina, Bodipudi Vineetha, Chandana Sai Kodali, Hema Sreelakshmi Guddeti, Sanjana Poladi, Chandana Priya Digumurthy, Jashika Mellamputi

**Affiliations:** 1 Internal Medicine, Apollo Institute of Medical Sciences and Research, Chittoor, IND; 2 Respiratory Medicine, University of Chester, Chester, GBR; 3 Medicine, Apollo Hospitals, Anantapur, IND

**Keywords:** anti-cyclic citrullinated peptide, heart failure, inflammatory markers, rheumatoid arthritis, tnf alpha inhibitors

## Abstract

Rheumatoid arthritis (RA) is a chronic inflammatory process involving the joints and the cartilage. Early diagnosis and treatment of RA are crucial to prevent further complications like heart failure (HF), cervical subluxation, membranous glomerulonephritis, and parenchymal lung disease. HF is the leading cause of death in various chronic illnesses. The pathophysiology of HF varies based on the type of illness. In this review, our main objective was to look into the association between RA and HF by examining the pathogenesis, risk factors, and treatment of HF in RA. We also discuss the role of the inflammatory markers associated with HF in the pathogenesis. The treatment of anti-inflammatory drugs in HF associated with RA is also discussed. We also address the risk factors associated with RA so that the development of complications like HF can be reduced in this patient population.

## Introduction and background

Rheumatoid arthritis (RA) is a common form of inflammatory arthritis with a global prevalence in all ethnic groups. The prevalence of RA is approximately 0.8-1.0% in Europe and the Indian subcontinent, with a female-to-male ratio of 3:1. The prevalence is lower in Southeast Asia (0.4%). RA is a complex disease with both genetic and environmental components. Cigarette smoking is an environmental risk factor. Genomic-wide association studies have detected nearly 100 loci associated with the risk of developing RA. The disease is characterized by infiltration of the synovial membrane with the lymphocytes, plasma cells, dendritic cells, and macrophages [1]. The incidence of RA is lower in women who actively take oral contraceptives (OCPs) compared to those who never took OCPs and those who took OCPs previously and stopped using them [2]. Risk is higher in women with subfertility and those in immediate postpartum periods after their first pregnancy [3]. A typical presentation involves pain, joint swelling, and stiffness affecting the small joints of the hands, feet, and wrists symmetrically. Clinical criteria, erythrocyte sedimentation rate (ESR), C-reactive protein (CRP), ultrasound or MRI, rheumatoid factor (RF), and anti-citrullinated peptide antibodies are useful in establishing the diagnosis of RA [4].

Heart failure (HF) refers to the clinical syndrome that develops when the heart cannot maintain adequate output or can do so only at the expense of elevated ventricular filling pressures [4]. The etiology of HF is linked to external factors (increased mechanical strength due to hypertension or valve pathology) or internal factors such as coronary artery disease [3]. The management of HF is based on several factors, whether acute or chronic. RA is one of the various risk factors for HF [5]. The higher expression of biomarkers such as interleukin-6 (IL-6), intercellular adhesion molecule (ICAM), and CRP, which leads to inflammation, fibrosis, and RAAS activation, is involved in the development of HF in patients with RA [5]. Hence, there is an increased risk of cardiovascular disease in patients with RA compared to the general population [6]. Therefore, there is a need for specialized care for patients with RA to decrease mortality due to chronic illnesses like cardiovascular disease [7]. Despite the high burden of HF in patients with RA, there are few studies about the risk factors, clinical presentation, and pathogenesis of RA in patients with HF. Hence, the main objective of the study is to determine the association between RA and HF and the pathogenesis of HF in patients with RA.

## Review

Methodology

We conducted a review by searching the PubMed and Google Scholar databases. We looked for studies published between January 1, 1990, and May 31, 2024. The keywords used to identify relevant articles included "rheumatoid arthritis and heart failure". We focused on studies evaluating the pathogenesis of HF in patients with RA, the association between RA and HF, and the treatment of HF in RA. Studies focusing on the clinical presentation of RA and the epidemiology of HF and RA were excluded. 

Rheumatoid arthritis and heart failure

A study by Nicola et al. reported that congestive heart failure is strongly associated with mortality among patients with RA [8]. RA is a chronic inflammatory condition that triggers auto-immunity in a genetically susceptible host by modifying host proteins through processes like citrullination so that they become immunogenic [2]. Citrullinated synovial proteins induce the production of RA-specific autoantibodies (anti-CCP), which in turn can induce the risk of heart disease [9,10]. Liang et al. conducted a population-based cohort study. They found that RF/antinuclear antibodies (ANA)/cyclic citrullinated peptide (CCP) is associated with an increased risk of HF in patients with RA [11]. The study by Lazúrová and Tomáš concluded that RA is linked to an increased risk of cardiovascular-related morbidity and mortality. Pro-inflammatory markers like IL-6, tumor necrosis factor-alpha (TNF-alpha), and CRP that are markedly increased in RA play a role in accelerating atherosclerosis and myocardial fibrosis development [12]. 

Ischemic heart disease (IHD) occurs due to decreased blood supply to the myocardial tissue. The main pathological process involved is atherosclerosis, which refers to the narrowing of arteries due to the formation of cholesterol plaques. Hence, all the mechanisms and processes that lead to the stabilization of atherosclerotic plaque are involved in the development of IHD. RA associated with specific genetic and immune mechanisms leads to the activation of CD-4 cells, which leads to the activation of macrophages and their recruitment to the site of inflammation and the production of pro-inflammatory markers (IL, TNF, and CRP) [13]. 

Low-density lipoproteins (LDL) pass through the endothelial cells by receptor-mediated endocytosis. TNF-alpha, which is a pro-inflammatory, leads to neutrophil accumulation through the activation of ICAM-1 on endothelial cells and L-selectin on neutrophils that lead to diapedesis of neutrophils into the blood vessel and the release of free radicals that react with the LDL. This leads to the formation of oxidized LDL that are phagocytosed by the neutrophils. This process attracts more neutrophils to the site and the engulfment of oxidized LDL. Then, all these cells combine to form a plaque that is covered by an endothelial lining called atherosclerotic plaque, which is the main pathological process of IHD. ILs, particularly IL-6, have a negative correlation with cholesterol metabolism in females, which in turn stabilizes atherosclerotic plaque and results in IHD. CRP is an acute-phase protein secreted by the liver and causes endothelial dysfunction that leads to the stabilization of atherosclerotic plaque and is involved in the development of IHD [14].

**Figure 1:**
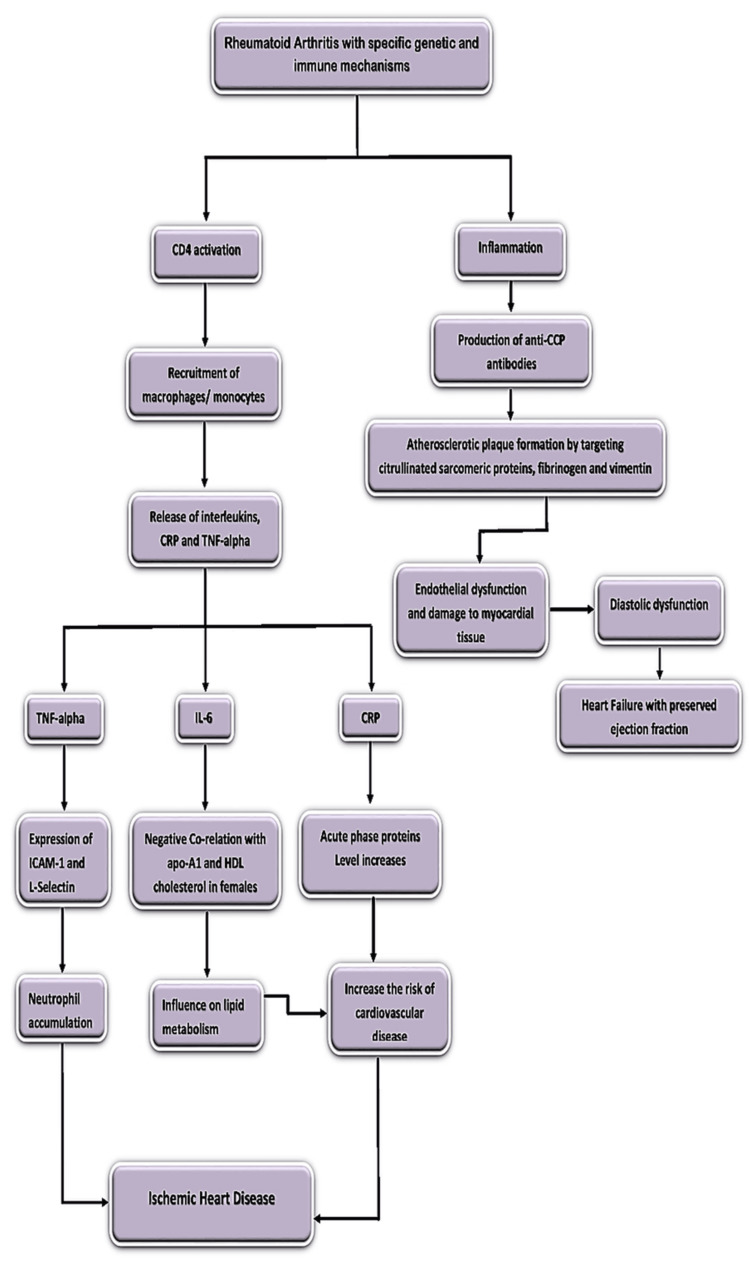
Pathogenesis of heart failure in rheumatoid arthritis Anti-CCP: anti-cyclic citrullinated peptide antibodies; Apo A-1: apoprotein A-1; CD-4: cluster of differentiation; CRP: C-reactive protein; HDL: high-density lipoprotein; ICAM: intercellular adhesion molecule; IL-6: interleukin-6; TNF-alpha: tumor necrosis factor-alpha Image credits: Dr. Deepika Korimerla

RA, which is a chronic inflammatory state, is also associated with the production of anti-CCP antibodies, which cause diastolic dysfunction by binding to specific proteins of cardiomyocytes that lead to myocardial remodeling and the development of HF with preserved ejection fraction [15,16]. This leads to elevated inflammation early in patients with RA that progresses to HF, particularly HF with preserved ejection fraction and not HF with reduced ejection fraction [17]. The pathogenesis of HF in RA is illustrated in Figure 1.

Kadier et al. conducted a cross-sectional study in a US population and a Mendelian randomization analysis in a European population and found an elevated prevalence of HF in the US population [18]. Park et al. conducted a longitudinal study in 2022. A total of 158 patients without clinical cardiovascular disease were enrolled and followed up for four to six years with echocardiography to assess for diastolic dysfunction. Baseline diastolic dysfunction was associated with RA disease activity [odds ratio (OR): 1.39; 95% confidence interval (CI): 1.02-1.90; p=0.034] [19]. Mantel et al. conducted a study to investigate the relative risk of HF and subtypes (ischemic and non-ischemic) in RA patients and found that in the new-onset RA cohort, the overall hazard ratio (HR) for subsequent ischemic and non-ischemic HF was between 1.22 and 1.27; hence, they concluded that non-ischemic HF is associated with an increased risk of RA severity [20]. 

McCoy et al. conducted a population-based cohort study in Minnesota to investigate the risk profiles, treatment, and outcomes of patients suffering from RA with myocardial infarction (MI) and matched MI patients without RA; they concluded that despite receiving similar treatment, MI patients with RA had worse long-term outcomes than MI patients without RA [21]. Nicola et al. conducted a population-based cohort study in 2006 in Minnesota, involving 575 patients with RA and 583 subjects without RA; after 30 years of follow-up, they found that RA is associated with a significant excess risk of congestive heart failure (HR: 1.87, 95% CI: 1.47-2.39). The risk was higher among patients with RA who were RF-positive (HR: 2.59, 95% CI: 1.95-3.43) compared to those who were RF-negative (HR: 1.28, 95% CI: 0.93-1.78) [22]. The studies exploring the association between RA and HF are summarized in Table 1.

**Table 1:** Studies exploring the association between rheumatoid arthritis and heart failure ACS: acute coronary syndrome; IHD: ischemic heart disease; LVEF: left ventricular ejection fraction; RA: rheumatoid arthritis

Study	Design	No. of participants	Population	Main conclusion
Park et al. (2022) [19]	Longitudinal study	158	RA patients without clinical cardiovascular disease	Diastolic dysfunction was prevalent in RA patients without clinical heart failure
Mantel et al. (2017) [20]	Case-control study	_	Patients with RA and without RA	Patients with RA are at increased risk of both ischemic and non-ischemic heart failure
McCoy et al. (2013) [21]	Population-based cohort study	231	Myocardial infarction patients with RA and myocardial patients without RA	Myocardial infarction patients with RA receiving similar treatment and cardioprotective medications had similar short-term outcomes compared to patients without RA
Nicola et al. (2005) [22]	Population-based cohort study	_	Patients with RA and patients without RA	Congestive heart failure rather than IHD appears to be an important contributor to the excess overall mortality among patients with RA
Wolfe et al. (2003) [23]	Community-based study	11,572	Patients with RA and patients with osteoarthritis	RA is associated with increased risk for cardiovascular and/or cerebrovascular disease, morbidity due to myocardial infarction, chronic heart failure, and probably cerebrovascular accident
Schau et al. (2014) [24]	Prospective cross-sectional study	157	Patients with RA	Heart failure was found elevated 4-6-fold in active RA and the predominantly prevalent type was diastolic heart failure with normal ejection factors
Mantel et al. (2015) [25]	Case-control study	762	Patients with ACS and without ACS	Risk factors for ACS in incident RA are clinical markers of inflammatory activity, and disease activity, whereas there was no association between RA and cardiovascular risk in RA
Faxén et al. (2023) [26]	Case control study	_	Patients with heart failure and without heart failure	RA was associated with heart failure with LVEF >40%

Treatment of heart failure in rheumatoid arthritis

Methotrexate is an anti-inflammatory drug used for the treatment of HF in patients with RA as it down-regulates T-cell activity, which is involved in the pathogenesis of RA and also in the development of atherosclerotic plaque instability and acute coronary syndromes [27]. Myasoedova et al. conducted a population-based cohort study to assess the influence of anti-rheumatic medications on the risk of HF in patients with RA and found that methotrexate use has been identified in the treatment of HF with RA [28]. Weisman et al. conducted a randomized double-blinded study to evaluate the safety of etanercept in patients with RA and concomitant comorbidities. Data from 535 patients were analyzed in the study. No etanercept-related increase in MI (3.7% placebo, 3-05% etanercept) or overall infections was observed; hence, they concluded that etanercept was generally well tolerated by RA patients with comorbidities [29].

TNF-alpha inhibitors are used to control the inflammatory process involved in the pathogenesis of HA in RA [30]. TNF receptor 1 (TNFR1) induces apoptosis and has negative ionotropic effects whereas TNFR2 promotes cell adhesion, angiogenesis, and cell survival, making TNFR1 and TNFR2 agonists potent agents in the management of HF in patients with RA, along with risk factor analysis [29]. Some patients with RA also receive other biological drugs that suppress IL-6 signaling and B-cells or targeted synthetic disease-modifying anti-rheumatic disease which inhibit Janus kinase activity and have decreased cardiovascular mortality among patients with RA in recent years [30]. The total cholesterol/HDLc ratio is a better predictor of CVD in patients with RA [31]. Hence lipid lipid-controlling drugs like statins, which stabilize the plaque, reduce inflammation, and decrease thrombogenicity, are being used along with disease-modifying drugs like tocilizumab to decrease cardiovascular mortality [31].

Giles et al. conducted a randomized control trial to assess the risk of major adverse cardiovascular events (MACE) in patients with RA treated with tocilizumab compared to those treated with TNF inhibitor etanercept and found that the estimated HR for the occurrence of MACE in the tocilizumab group relative to the etanercept group was 1.05 (95% CI: 0.77-1.43); they concluded that there is an increased risk for the occurrence of MACE with tocilizumab compared to etanercept [32]. Dixon et al. conducted a prospective observational study comparing MI rates in patients with RA treated with anti-TNF-alpha and active RA patients treated with disease-modifying anti-rheumatic drugs (DMARDS) and found that the risk of MI is markedly reduced in those who respond to anti-TNF-alpha therapy compared to non-responders [33]. A study conducted by Low et al. involving patients with RA who are starting therapy with TNF inhibitors and another group treated with DMARDS found that exposure to TNF inhibitors does not appear to influence the occurrence of ischemic stroke in patients with RA [34]. The studies exploring the relationship between anti-inflammatory drugs and HF are summarized in Table 2.

**Table 2:** Studies exploring the treatment of heart failure in rheumatoid arthritis Anti-TNF-alpha: anti-tumor necrosis factor-alpha; CHF: chronic heart failure; DMARDs: disease-modifying anti-rheumatic drugs; RA: rheumatoid arthritis; TNF inhibitor: tumor necrosis factor inhibitor

Study	Design	No. of participants	Population	Main conclusion
Weisman et al.(2007) [29]	Randomized double-blind study	535	RA patients with comorbidity	Etanercept was well tolerated by RA patients with comorbidities and the incidence of myocardial infarction was not increased by etanercept treatment
Giles et al. (2020)[32]	Randomized controlled study	3,080	Patients with RA treated with tocilizumab and patients with RA treated with TNF inhibitor etanercept	There is an increased risk of major adverse cardiovascular events in patients treated with tocilizumab than with etanercept
Dixon et al. (2007) [33]	Prospective observational study	10,840	Patients with RA treated with anti-TNF-alpha and patients with RA treated with DMARDs	There is a reduction in the incidence of MI in patients with RA who respond to anti-TNF-alpha therapy
Low et al. (2006) [34]	Cohort study	_	Patients with RA treated with TNF inhibitors and patients with RA treated with DMARDs	Exposure to TNF inhibitor therapy does not influence the occurrence of ischemic stroke in patients with RA
Hu et al. (2021) [35]	Meta-analysis	_	Cohort studies and randomized controlled trials till January 2021 to assess the association between DMARDs and the risk of cardiovascular events in patients with systemic inflammatory conditions were included	Anti-inflammatory therapies including biological DMARDs decrease the risk of cardiovascular disease
Buch et al. (2024)[36]	Randomized controlled trial	_	Patients with RA treated with tofacitinib and patients with RA treated with TNF inhibitor therapy	The risk of ischemic cardiovascular and heart failure did not appear different between tofacitinib and TNF inhibitors
Heslinga et al. (2015) [37]	Systematic review	_	Analysis of 54 studies published till 2013 investigating the effect of blocking therapy on the occurrence and risk of CHF in patients with RA	In patients with concomitant heart failure, treatment with TNF blocking therapy is not contraindicated

Limitations

This review has a few limitations. We used only a few databases such as PubMed to find relevant articles. There are limited studies on the definitive treatment for HF in RF and further studies are warranted.

## Conclusions

This review explores the association between RA and HF, a potential complication of RA. Our findings show that the chronic inflammatory state in RA is a risk factor for the development of cardiovascular disease. Our results also point to the role that inflammatory markers in RA patients play in the development of HF, in both ischemic and non-ischemic types. The review also highlights that the early diagnosis of RA and evaluation of inflammatory markers like anti-citrullinated peptides, ESR, and CRP are crucial, and early treatment with anti-inflammatory drugs in RA patients can reduce the development of complications like HF. We believe that our findings can help raise awareness among clinicians in correlating RA with HF, by elaborating on the pathophysiology and various treatment modalities that are being used in coexisting diseases. Our findings reveal that TNF-alpha inhibitors are the most commonly used drugs to prevent the progression of inflammation in HF in RA patients. Further research into the association between RA and HF and exploration of potential treatment strategies can help devise a tailor-made protocol for the optimal management of this patient population.
